# Experimental colitis reduces microglial cell activation in the mouse brain without affecting microglial cell numbers

**DOI:** 10.1038/s41598-019-56859-0

**Published:** 2019-12-27

**Authors:** Hoda M. Sroor, Ahmed M. Hassan, Geraldine Zenz, Paulina Valadez-Cosmes, Aitak Farzi, Peter Holzer, Amany El-Sharif, Fatma Al-Zahraa M. Gomaa, Julia Kargl, Florian Reichmann

**Affiliations:** 1 Research Unit of Translational Neurogastroenterology, Division of Pharmacology, Otto Loewi Research Centre for Vascular Biology, Immunology and Inflammation, Medical University of Graz, Graz, Austria; 2 Division of Pharmacology, Otto Loewi Research Centre for Vascular Biology, Immunology and Inflammation, Medical University of Graz, Graz, Austria; 3 Microbiology and Immunology Department, Faculty of Pharmacy-Girls, Al-Azar University, Cairo, Egypt; 4 Pharmacognosy and Medicinal Herbs Department, Faculty of Clinical Pharmacy, Al-Baha University, Al-Baha, Saudi Arabia

**Keywords:** Inflammatory bowel disease, Microglia, Neuroimmunology

## Abstract

Inflammatory bowel disease (IBD) patients frequently suffer from anxiety disorders and depression, indicating that altered gut-brain axis signalling during gastrointestinal inflammation is a risk factor for psychiatric disease. Microglia, immune cells of the brain, is thought to be involved in a number of mental disorders, but their role in IBD is largely unknown. In the current work, we investigated whether colitis induced by dextran sulphate sodium (DSS), a murine model of IBD, alters microglial phenotypes in the brain. We found that colitis caused a reduction of Iba-1 and CD68 immunoreactivity, microglial activation markers, in specific brain regions of the limbic system such as the medial prefrontal cortex (mPFC), while other areas remained unaffected. Flow cytometry showed an increase of monocyte-derived macrophages during colitis and gene expression analysis in the mPFC showed pronounced changes of microglial markers including cluster of differentiation 86 (CD86), tumour necrosis factor-α, nitric oxide synthase 2, CD206 and chitinase-like protein 3 consistent with both M1 and M2 activation. Taken together, these findings suggest that experimental colitis-induced inflammation is propagated to the brain altering microglial function.

## Introduction

Inflammatory bowel diseases (IBDs) are chronic inflammatory diseases of the gastrointestinal tract. The 2 major IBD entities, Crohn’s disease (CrD) and ulcerative colitis (UC), are chronic, relapsing maladies with both periods of active disease and remission. During disease exacerbations, IBD patients show a wide spectrum of symptoms including bloody diarrhoea and pain, severely reducing their quality of life^1,2^. Apart from gastrointestinal symptoms, accumulating evidence suggests that both CrD and UC patients have an increased risk for psychiatric disorders^3–5^. There is, for example, an increased prevalence of anxiety disorders and depression, which is particularly evident during periods of active disease^6^, indicating that altered gut-brain axis signals in the course of gastrointestinal inflammation influence brain function.

Dextran sulphate sodium (DSS)-induced colitis, an animal model of IBD^7,8^, has been shown to disrupt emotional-affective behaviour in rodents, effects which are significantly modulated in the presence of psychological stress^9–12^. These behavioural changes are accompanied by altered neuronal activation in the corticolimbic system^13^, increased brain excitability^12^ as well as brain region-specific changes in neuropeptide and inflammatory marker gene expression^9,11^, demonstrating that gastrointestinal inflammation alters neuronal function. In contrast, the effect of experimental colitis on non-neuronal cells, such as microglia, is less well known. Microglia, immune cells of the brain parenchyma, is unique in their transcriptional programing and surface marker expression and their activity can be altered by various diseases^14–16^. In addition, they are essential for the translation of peripheral immune signals to the brain to alter brain function^17^. Accordingly, the aim of the current work was to characterize the effects of experimental colitis on microglial cells in limbic brain areas relevant for emotional-affective behaviour and to investigate its modulation by psychological stress. To this end, we have used the microglial markers ionized calcium-binding adapter molecule 1 (Iba-1) and cluster of differentiation (CD) 68 to identify limbic brain areas that show microglial alterations in response to gastrointestinal inflammation or stress. We then evaluated microglial polarization in the medial prefrontal cortex (mPFC), a brain area which showed pronounced Iba-1 alterations using markers for M1 status (CD86, nitric oxide synthase 2 (Nos2), tumour necrosis factor-alpha (TNF-α), interleukin-1beta (IL-1β)) as well as M2 status (CD206, arginase 1 (Arg1), chitinase-like 3 (Chil3)). Finally, we quantitated colitis-induced changes in brain microglial and monocyte-derived macrophage cell numbers using flow cytometry. We hypothesized that experimental colitis and psychological stress are able to alter microglial phenotype in various limbic brain areas.

## Results

### Water avoidance stress (WAS) does not induce or modify colonic inflammation

As previously described^13^, mice were treated with 2% (w/v) DSS in drinking water for 7 days to induce mild colitis while control mice received plain drinking water. At the end of this treatment period, half of the mice from each group were exposed to a 30-minute session of WAS, a model of mild psychological stress^13,18^, while the other half remained unstressed (Table 1). In line with previous findings^13,19^, disease activity index (DAI; DSS main effect: F_(1, 28)_ = 195.6; p < 0.001)) and the colonic content of myeloperoxidase (MPO), a marker of colonic leukocyte infiltration, was significantly increased in DSS-treated mice (DSS main effect: F _(1_, _28)_ = 95.5; p < 0.001), which confirms the presence of colitis in response to DSS. In contrast, WAS itself did not alter DAI and MPO levels nor modify the DSS-induced DAI and MPO rises (Table 1).

**Table 1:** Study groups and treatment effects of 2% dextran sulphate sodium (DSS) compared to normal drinking water (VEH) and water avoidance stress (WAS) compared to basal conditions (CO) on colonic myeloperoxidase (MPO) content and disease activity index (DAI).

groups	treatment	exposure to stress	MPO (µg/g; mean ± SEM)	DAI (mean ± SEM)
VEH + CO	normal tap water	no WAS	1.0 ± 0.18	0.9 ± 0.30
VEH + WAS	normal tap water	30 min WAS	1.2 ± 0.37	1.1 ± 0.23
DSS + CO	2% DSS (w/v) for 7 days	no WAS	31.2 ± 11.28***	5.9 ± 0.23***
DSS + WAS	2% DSS (w/v) for 7 days	30 min WAS	42.2 ± 9.94 ***	5.4 ± 0.50***

Data are presented as means ± SEM, n = 8 per group. Two-way analysis of variance (ANOVA), ***p < 0.001 DSS main factor effect.

### DSS-induced colitis, but not WAS, reduces Iba-1 and CD68 immunoreactivity in a brain region-specific manner

**Figure 1:**
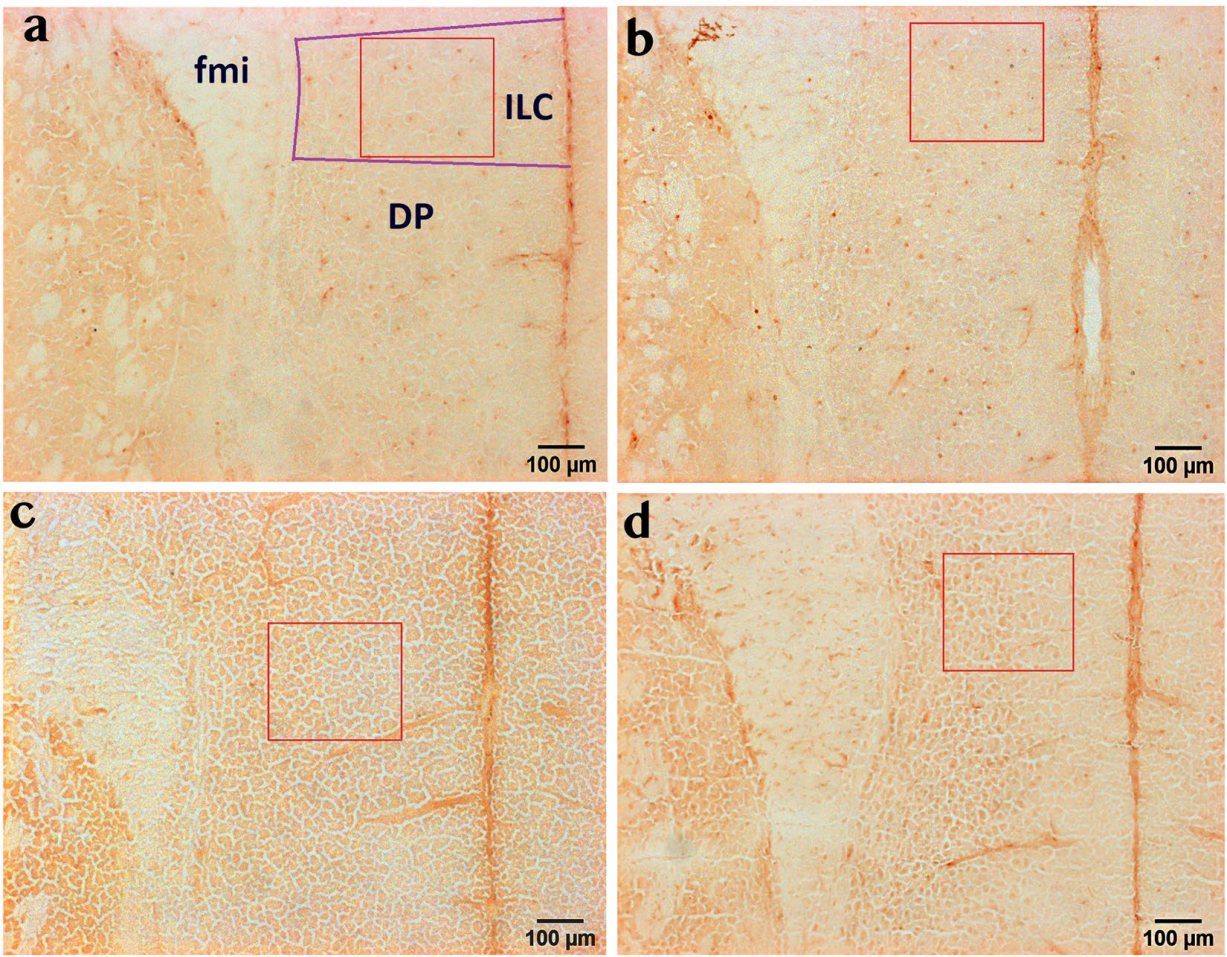
Colitis decreases Iba-1 immunoreactivity in the infralimbic cortex (ILC), independently of stress exposure. Panels a-d show representative images of Iba-1 immunohistochemical micrographs for the 4 treatment groups under study: VEH/CO (**a**), VEH/WAS (**b**), DSS/CO (**c**) and DSS/WAS (**d**); The selected counting area within the region of interest is indicated by a red rectangle (300 × 300 µm). Abbreviations: DP-dorsal peduncular cortex, fmi-forceps minor corpus callosi, ILC-infralimbic cortex.

**Figure 2:**
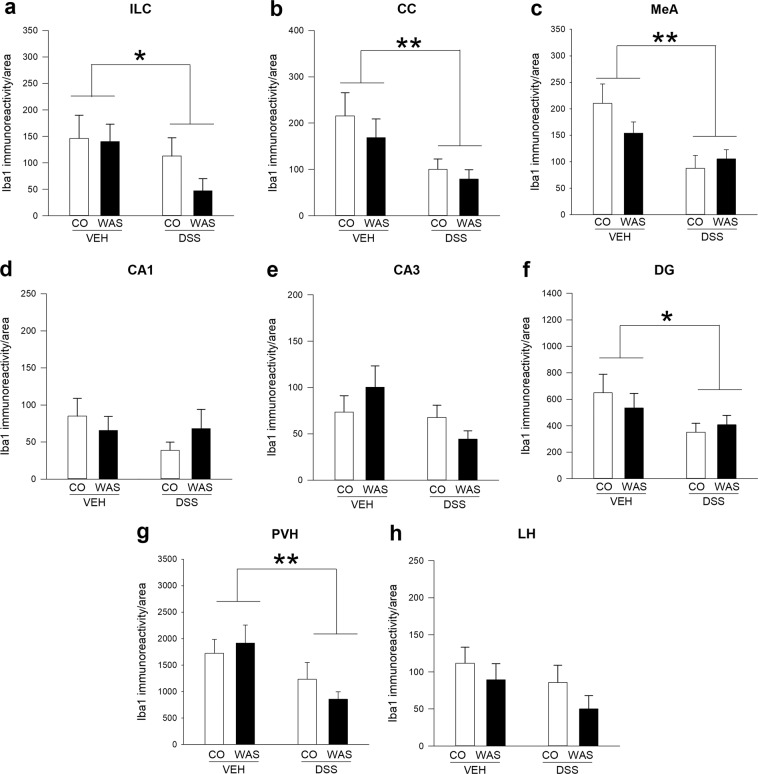
Brain region-specific effects of colitis and water avoidance stress (WAS) on Iba-1 immunoreactivity in the limbic system. DSS-induced colitis decreases Iba-1 immunoreactivity in the infralimbic cortex (ILC; **a**), cingulate cortex (CC; **b**) and medial amygdala (MeA; **c**), while WAS had no effect. In the hippocampus, both colitis and WAS failed to alter Iba-1-immunoreactivity in Cornu ammonis area 1 (CA1; **d**) and Cornu ammonis area 3 (CA3; **e**), but colitis reduced Iba-1 signals in the dentate gyrus (DG; **f**) independently of stress exposure. In the hypothalamus colitis decreased Iba-1 levels in the paraventricular nucleus of the hypothalamus (PVH; **g**) independently of stress exposure, but both colitis and WAS did not alter Iba-1 immunoreactivity in the lateral hypothalamus (LH; **h**). Two-way analysis of variance (ANOVA), **p < 0.01, *p < 0.05 DSS main factor effect.

To assess the impact of colitis and stress on brain microglial cells, we investigated Iba-1 and CD68 immunoreactivity, microglial activation markers^17^, in selected brain regions relevant for emotional-affective behaviour and stress processing, including medial prefrontal cortex (mPFC), hippocampus, amygdala and hypothalamus^20,21^. We found that within the mPFC, DSS-induced colitis significantly decreased Iba-1 immunoreactivity both in the infralimbic cortex (ILC) (F_(1, 23)_ = 4.4; p = 0.05) and in the cingulate cortex (CC) (F_(1, 24)_ = 8.4; p = 0.008), attesting to a marked effect of experimental colitis on this brain region (Fig. 1; Fig. 2a,b). In contrast, WAS did not alter Iba-1 immunoreactivity in the mPFC or modify the colitis-induced reduction of this marker. Similarly, colitis decreased Iba-1 immunoreactivity in the medial amygdala (MeA) (F_(1, 24)_ = 10.7; p = 0.003), with no significant WAS effect and no significant interaction between these two factors (Fig. 2c). In the hippocampus, the effects of colitis were less pronounced, as no significant difference in Iba-1 immunoreactivity between the treatment groups could be identified in the Cornu Ammonis area 1 (CA1) and Cornu Ammonis area 3 (CA3) region (Fig. 2d,e). Interestingly, however, DSS-induced colitis significantly reduced Iba-1 immunoreactivity (F_(1, 24)_ = 4.4; p = 0.046) in the dentate gyrus (DG) (Fig. 2f), suggesting distinct effects on hippocampal subregions. Like in the mPFC and MeA, WAS had no effect on hippocampal Iba-1 immunoreactivity and did not modify colitis-induced Iba-1 changes (Fig. 2d–f). Finally, in the hypothalamus, DSS-induced colitis significantly reduced Iba-1 levels in the paraventricular nucleus of the hypothalamus (PVH) (F _(1, 24)_ = 7.9; p = 0.01), the prime element of the hypothalamo-pituitary-adrenal axis and the stress output system (Fig. 2g), but did not influence Iba-1 levels in another hypothalamic region, the lateral hypothalamus (Fig. 2h). Similar to all other brain regions, WAS failed to influence Iba-1 immunoreactivity in any of the hypothalamic regions examined (Fig. 2g,h).

**Figure 3:**
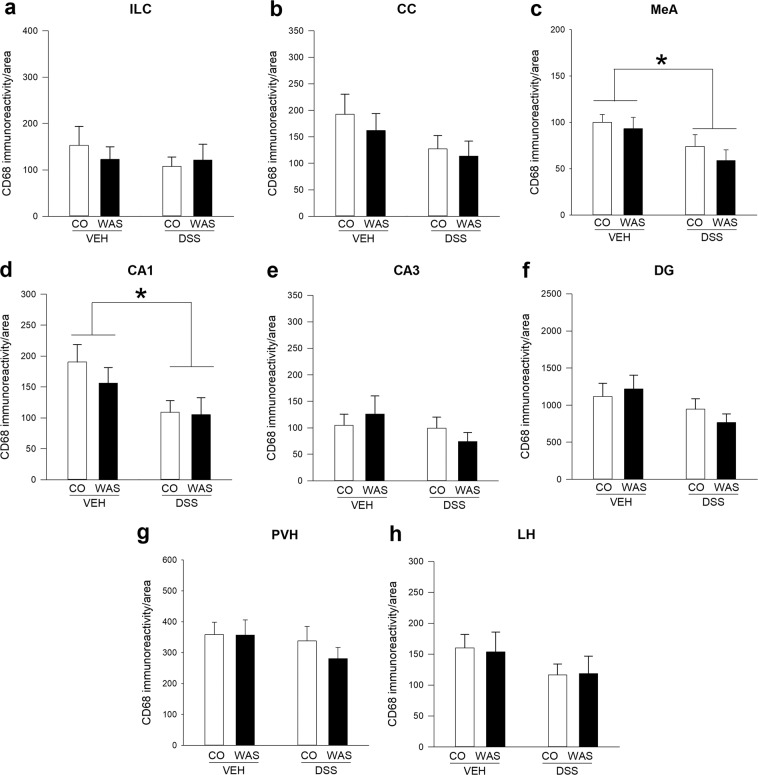
Brain region-specific effects of colitis and water avoidance stress (WAS) on CD68 immunoreactivity in the limbic system. Both DSS-induced colitis and WAS did not alter CD68 immunoreactivity in the infralimbic cortex (ILC; **a**) and cingulate cortex (CC; **b**), but colitis reduced CD68 levels in the medial amygdala (MeA; **c**) and Cornu ammonis area 1 (CA1; **d**) independently of stress exposure. WAS and colitis failed to alter CD68 immunoreactivity in other hippocampal and hypothalamic brain regions including Cornu ammonis area 3 (CA3; **e**), dentate gyrus (DG; **f**), paraventricular nucleus of the hypothalamus (PVH; **g**) and lateral hypothalamus (LH; **h**). Two-way analysis of variance (ANOVA), *p < 0.05 DSS main factor effect.

**Figure 4:**
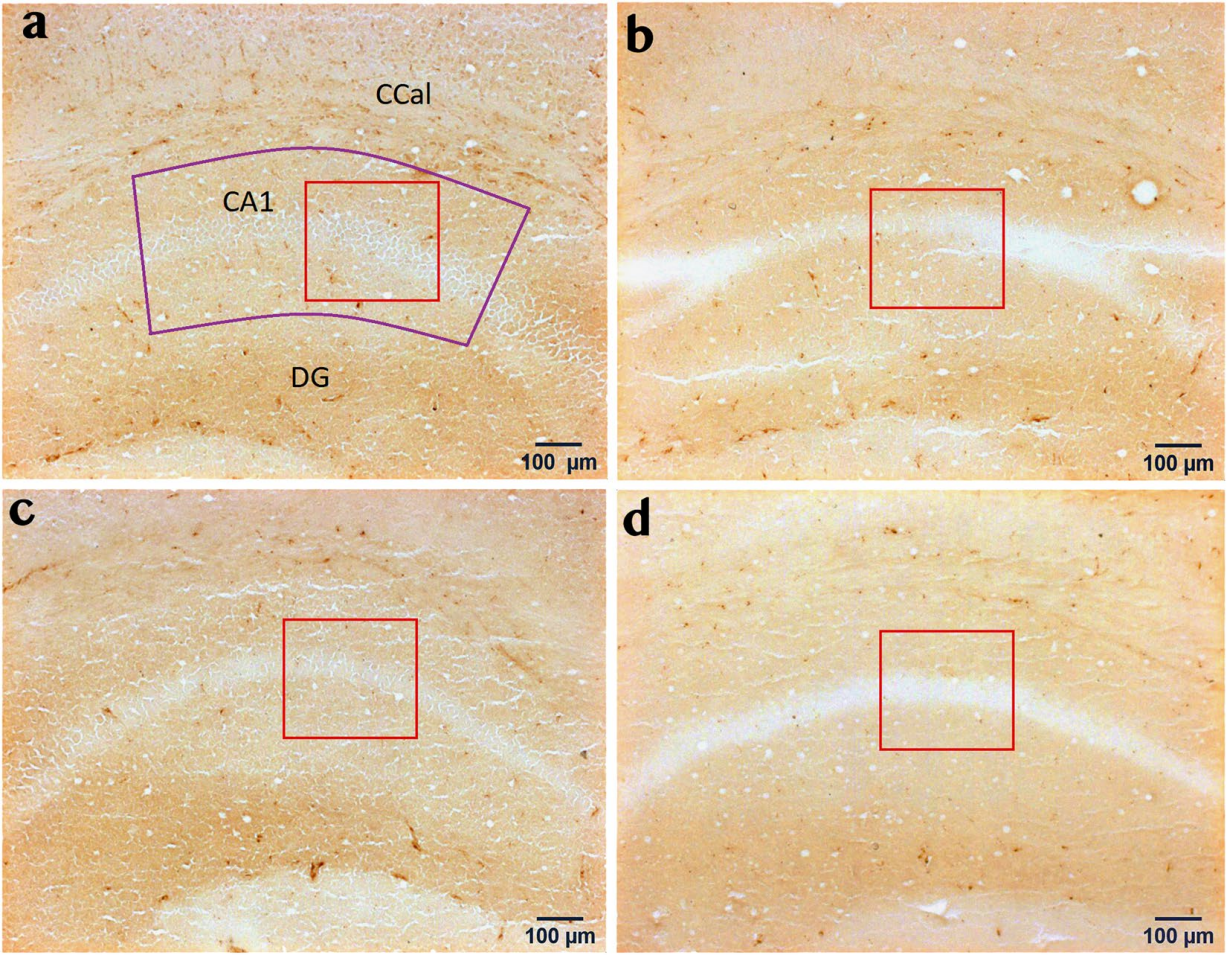
Colitis decreases CD68 immunoreactivity in the Cornu ammonis area 1 (CA1), independently of stress exposure. Panels a–d show representative images of CD68 immunohistochemical micrographs for the 4 treatment groups under study: VEH/CO (**a**), VEH/WAS (**b**), DSS/CO (**c**) and DSS/WAS (**d**); The selected counting area within the region of interest is indicated by a red rectangle (300 × 300 µm). Abbreviations: CA1- Cornu ammonis area 1, CCal-corpus callosum, DG-dendate gyrus.

CD68 immunoreactivity was also altered by colitis in a brain region-specific manner. Specifically, colitis did not influence CD68 immunoreactivity in the ILC (Fig. 3a), but tended to decrease CD68 levels in the CC (Fig. 3b; F_(1, 25)_ = 3.199; p = 0.086). Similar to Iba-1, colitis significantly decreased CD68 immunoreactivity in the MeA (F_(1, 25)_ = 7.241; p = 0.013) with no significant WAS effect and no significant interaction between these two factors (Fig. 3c). We also detected a colitis-induced reduction of CD68 immunoreactivity in the hippocampal CA1 region (F_(1, 24)_ = 6.799; p = 0.015; Fig. 4) and a trend towards reduced CD68 immunoreactivity in the DG (F_(1, 25)_ = 3.781; p = 0.063), but not in the hippocampal CA3 region, again similar to the Iba-1 results (Fig. 3d–f). In contrast, hypothalamic CD68 expression in the PVH and LH (Fig. 3g,h) was not affected by colitis. WAS did not alter CD68 immunoreactivity in any of the investigated brain regions (Fig. 3).

### DSS-induced colitis has pronounced effects on both M1 and M2 microglial markers in the mPFC

**Figure 5:**
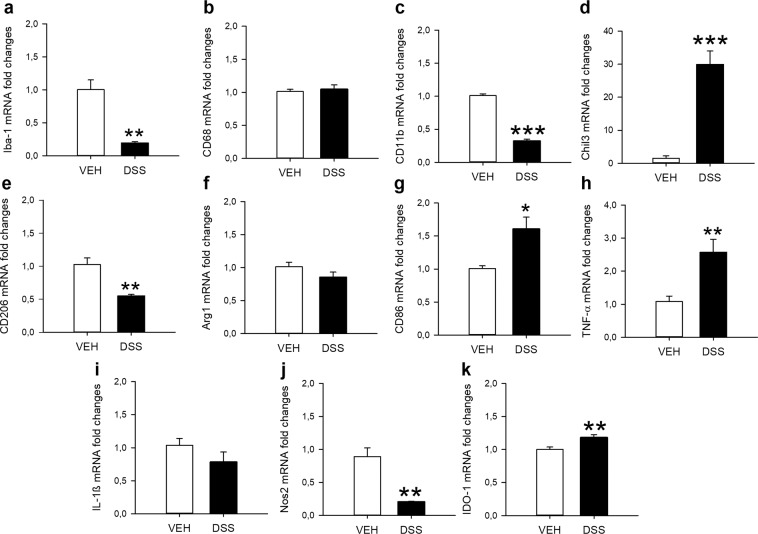
Colitis induces pronounced changes in microglial marker expression in the medial prefrontal cortex. Panels a-k show relative mRNA expression levels of ionized calcium-binding adapter molecule 1 (Iba-1; **a**), cluster of differentiation 68 (CD68; **b**), cluster of differentiation 11b (CD11b; **c**), chitinase-like protein 3 (Chil3; **d**), cluster of differentiation 206 (CD206; **e**), arginase 1 (Arg1; **f**), cluster of differentiation 86 (CD86; **g**), tumour necrosis factor-α (TNF-α; **h**), interleukin 1β (IL-1ß; **i**), nitric oxide synthase 2 (Nos2; j) and indoleamine 2,3-dioxygenase 1 (IDO-1; k). The data presented are means + SEM, n = 7–8/group; t-tests, *p < 0.05, **p < 0.01, ***p < 0.001 vs. VEH.

In order to characterize the microglial profile in a brain area in which Iba-1 immunoreactivity was decreased during DSS-induced colitis in more detail, we evaluated selected markers of microglial phenotype and inflammation in the mPFC by qPCR. In line with the immunohistochemical data, DSS-induced colitis significantly reduced *Iba-1* gene expression (t_6.3_ = 5.5; p = 0.001), but did not change *CD68* expression within this brain area (Fig. 5a,b). *CD11b*, a marker expressed by both resting and activated microglia, was decreased as well (t_13_ = 17.85; p < 0.001; Fig. 5c). Colitis also changed the expression of M1 (pro-inflammatory) and M2 (anti-inflammatory) microglial markers indicating complex effects on this cell population. The most pronounced effect was detected for *chitinase-like protein 3* (*Chil3*), a marker for the M2 (anti-inflammatory) microglial phenotype, which showed an almost 30 fold higher expression in colitis samples (t_6.345_ = 6.738; p < 0.001; Fig. 5d). In contrast, the expression of *CD206*, another M2 marker, was decreased (t_7.9_ = 4.7; p = 0.002; Fig. 5e), while the expression of *arginase 1* (*Arg1*), a third M2 marker, was unchanged (Fig. 5f). M1 (pro-inflammatory) marker expression was also either increased, unchanged or decreased by colitis. Specifically, colitis enhanced mRNA levels of *CD86* (t_7.8_ = −3.2; p = 0.013; Fig. 5g) and *TNF-α* (t_9.3_ = −3.4; p = 0.006; Fig. 5h), did not change *IL-1β* expression (Fig. 5i), but decreased *Nos2* expression (t_7.092_ = 5.237; p = 0.001; Fig. 5j). In addition, DSS-induced colitis increased the expression of the indoleamine 2,3-dioxygenase 1 *(IDO-1)* gene (t_14_ = −3.2; p = 0.007) (Fig. 5k), which has been suggested as an important link between microglial activation and behaviour^22–24^.

### DSS-induced colitis does not alter the amount of microglial cells, but increases the number of monocyte-derived macrophages

**Figure 6:**
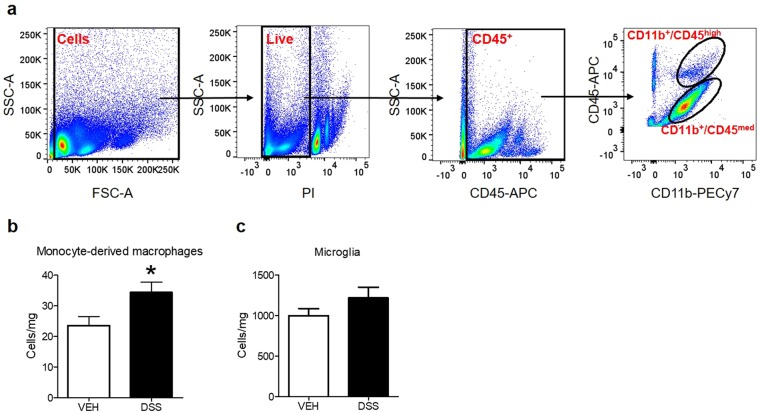
Colitis increases the number of monocyte-derived macrophages in the brain. (**a**) Representative polychromatic dot plots demonstrating the gating strategy employed to identify microglia and monocyte-derived macrophages in the brains of mice. Starting at the top left, a size gate was applied followed by gating on live (PI) and CD45+ cells. Finally, microglia cells were defined as CD11b+/CD45^med^ and monocyte-derived macrophages were identified as CD11b+/CD45^high^ (**b**,**c**). Colitis increased the number of monocyte-derived macrophages, but did not alter the number of microglial cells. Cell counts normalized to tissue weight. Data are presented as means + SEM, n = 10/group; t-tests, *p < 0.05, vs. VEH.

Finally, we used flow cytometry to evaluate a potential effect of colitis on the number of microglial cells or monocyte-derived macrophages. To analyse these 2 cell populations, we used the surface markers CD45 and CD11b. As previously described^25^, microglia was identified as CD11b^+^/CD45^med^ cells, while monocyte-derived macrophages were identified as CD11b^+^/CD45^high^ cells (Fig. 6a). Analysis revealed a significant increase of monocyte-derived macrophages during DSS-induced colitis (t_18_ = 2.424; p = 0.026; Fig. 6b), but no change in absolute cell numbers of microglia (Fig. 6c)

## Discussion

Given the accumulating evidence of altered gut-brain axis signalling in the course of visceral inflammation^26,27^ and the well-established role of microglia in response to peripheral immune challenge and stress^17,28–30^, the current study set out to investigate whether DSS-induced colitis, an animal model of IBD, and WAS, a mild psychological stressor, would alter the brain’s microglia phenotype. Indeed, colitis caused a reduction in the immunoreactivity of the microglial markers Iba-1 and CD68 in the limbic system, whereas WAS had no effect. Gene expression analysis in the mPFC, a brain area that showed a particularly strong reduction in Iba-1 immunoreactivity in response to colitis, confirmed these findings and revealed pronounced effects on microglial polarization markers. In addition, flow cytometry showed a rise of monocyte-derived macrophages in the brains of colitis animals.

Peripheral immune challenge with lipopolysaccharide (LPS), injection of bacteria or bile duct ligation is known to elicit transient neuroinflammation, sickness behaviour, anhedonia and microglial activation as revealed by increased Iba-1, CD68 or CD11b immunoreactivity and increased gene expression of M1 (pro-inflammatory status) microglial markers including IL-1β, IL-6, TNF-α, Nos2 and CD86^17,31–33^. Microglia appears to play a pivotal role in these effects because minocycline, which selectively inhibits M1 polarization of microglia^34^, facilitates the recovery of mice from LPS-induced sickness behaviour, blocks LPS-induced anhedonia and attenuates the upregulation of pro-inflammatory cytokines induced by LPS^35^. In addition, microglial inhibition also blocks sickness behaviour and the reduction of brain catecholamines induced by peripheral *E*. *coli* injection^36^.

Here, we investigated the role of microglia during DSS-induced colitis, an animal model of IBD. Given that DSS-induced colitis alters emotional-affective behaviour^9,11,12^ and changes stress-induced neuronal activation in the limbic system^13^, we evaluated its effects on microglial cells in several limbic brain areas. Largely paralleling previous findings of reduced neuronal activation during colitis^13^, Iba-1 immunoreactivity was decreased in ILC, CC, DG, PVH, and MeA, suggesting that colitis affects microglia in many brain areas. In addition, we measured immunoreactivity of the microglial activation marker CD68 in these areas and detected similar colitis-induced reductions in MeA and CA1, suggesting reduced microglial activation during colitis. Interestingly, however, the effects of colitis on Iba-1 and CD68 immunoreactivity are not identical. A potential explanation is that although Iba-1 and CD68 are both widely used as microglial activation markers, they can be expressed in different microglial cells and react to inflammatory stimuli in a different manner^37^. In addition, a recent study showed that different populations of microglial cells are Iba-1 and CD68 immunoreactive under control conditions and in age-associated deep subcortical white matter lesions^38^. Moreover, not every brain area examined appears to be affected to the same extent, given that neither the Iba-1 nor the CD68 profile of some brain areas (CA3, LH) changed during colitis. Brain region-specific differences in blood-brain-barrier integrity or microglial diversity might account for these findings^39–41^.

Reduced microglial activation during DSS colitis, a peripheral model of inflammation, is surprising, given many studies reporting increased activation under various pathological conditions^17,42,43^. However, the effects of colitis on microglial activation is much less well known and the type, extent and mechanism of CNS inflammation under these conditions needs further investigation. A previous study using 2,4,6-trinitrobenzene sulfonic acid (TNBS)-induced colitis, another model of experimental colitis, reported an increase of Iba-1-positive hippocampal microglia 4 days after TNBS administration in rats^44^. This study appears to be in conflict with the current work, because here we found that administration of DSS for 7 days in mice decreases Iba-1 immunoreactivity in the DG. However, there are several potential explanations for this discrepancy. Most importantly, the experimental colitis model used in the two studies involves significantly different pathophysiological mechanisms^45^. There are also differences in the time points used to examine the microglial profile after induction of colitis, analysis methodology and species used (rats vs. mice), all of which may potentially contribute to these diverse findings. Further work, for example using infectious colitis models, is needed to better understand the role of hippocampal microglia in different animal models of colitis.

To further characterize the central immunological response to DSS colitis, we asked whether the number of microglial cells and monocyte-derived macrophages in the brain is altered by colitis. While we did not detect any colitis effect on microglial cell numbers, the number of monocyte-derived macrophages was higher in brain samples of animals with colitis. These cells are either perivascular macrophages or entering the brain via the blood stream^25^, suggesting enhanced brain infiltration of immune cells during colitis potentially causing inflammation-mediated behavioural and neurobiological effects.

Analysis of microglial marker expression in the mPFC showed pronounced alterations as well. *Iba-1* mRNA levels were decreased and *CD68* expression was unaltered in mice with DSS colitis, confirming the immunohistochemical findings at the transcriptional level in this brain area. Interestingly, these data are in line with a recent study describing reduced Iba-1 and CD68 staining in the experimental autoimmune encephalomyelitis (EAE) animal model^46^, which has been interpreted as a state of microglial “deactivation” by the authors. Analysis of M1 and M2 microglial status markers revealed complex effects of colitis. The proposed physiological function of the M1 microglial phenotype is to defend tissues against invading pathogens, which is characterized by the production of pro-inflammatory cytokines such as TNF-α and IL-1β and the expression of several surface markers, which include CD86. However, M1 over-activation may lead to neuronal damage through formation of pro-inflammatory cytokines, nitric oxide, and reactive oxygen species^42^. On the other hand, the M2 phenotype antagonizes the pro-inflammatory response and is characterized by production of anti-inflammatory cytokines such as IL-4, IL-13, IL-10 and the surface marker CD206^15,43^.

The most prominent change was seen for Chil3, a heparin-binding lectin and M2 marker^47^, with higher expression levels during colitis. This protein is highly expressed in regeneration-supporting microglia^48^, which might suggest the occurrence of regenerative processes during the acute phase of colitis. On the other hand, Chil3 is expressed by inflammatory macrophages^49^ and thus might be related to the detected rise of infiltrating macrophages in this study. Another interesting point is the comparison of *Arg1* and *Nos2* expression between the treatment groups. Both enzymes use the same substrate, arginine, and therefore can outcompete each other. The lower *Nos2* expression in the DSS group might suggest a shift of arginine metabolism towards Arg1 during colitis resulting in higher levels of the wound healing-promoting polyamines, proline and ornithine, and lower levels of nitric oxide and free radicals, consistent with M2 activation^47^.

However, we also found evidence for M1 activation. Specifically, the mRNA levels of the anti-inflammatory M2 status marker *CD206* were decreased, whereas markers of the pro-inflammatory M1 microglia status including *CD86* and *TNF-α* were increased. In line with the latter findings, Silverman *et al*.^50^ reported that LPS suppresses the mRNA expression of *Iba-1*, but increases the mRNA expression of *IL-1β* and *IL-6* together with morphological changes in microglia^50^. The authors suggested that the suppression of *Iba-1* mRNA might act as a stop signal to prevent microglial overactivation in response to LPS, which might be the case during colitis as well. Notably, experimental colitis did not increase *IL-1ß* expression in the mPFC, despite increasing *TNF-α* and *CD86* expression. This is in line with Riazi *et al*.^44^. who reported an experimental colitis-induced rise of hippocampal *TNF-α*, but not *IL-1ß* gene expression. These colitis-related findings differ, however, from the effect of LPS, which elevated both IL-1ß and TNF-α^51^, suggesting that colitis activates brain cytokines in a manner different from LPS. Taken together, we did not see a clear shift towards M1 or M2 microglial phenotype in the current study, since both the expression of M1 and M2 markers was either increased, unaltered or decreased by colitis. This might indicate that a number of different microglia subpopulations are activated in parallel under our conditions, or as recently proposed^52^ that the microglia in this study might show a mixed phenotype at the single cell level, expressing both M1 and M2 markers. Future work is needed to test these hypotheses.

We also observed increased *IDO-1* gene expression in the mPFC of mice with colitis. IDO-1 is expressed in microglia, and an overactivation of this enzyme shifts tryptophan metabolism to the kynurenine pathway instead of the serotonin pathway^53^. Interestingly, it has been proposed that activation of the kynurenine pathway of tryptophan metabolism is an important link between microglial activation and behaviour^23,24^. In particular, induction of IDO-1 activity and the subsequent activation of the kynurenine pathway are involved in the behavioural impairments induced by LPS^24^ and Bacillus Calmette–Guérin (BCG)^23^. Our results suggest that a similar activation of the kynurenine pathway is also happening during experimental colitis. It should however be noted that the detected effect size is small and that further experiments are needed to determine biological significance.

In rodents, microglia is not only activated in response to immune challenges but also in response to acute and chronic psychological stress^28,54,55^. Furthermore, the reactivity of microglia to psychological stress is potentially involved in the pathophysiology of stress-related mental disorders including major depression^55,56^. Considering these findings, it is worth noting that stress is also a major factor determining disease course and causing exacerbations in patients suffering from IBD^57,58^. In addition, functional magnetic resonance imaging studies showed altered neuronal activation patterns in the brain of IBD patients in response to stress^59,60^, suggesting aberrant stress coping mechanisms. WAS has been frequently used to study stress effects in animal models of IBD^9,11,61^ as this paradigm is able to alter neuronal activation during colitis and in healthy animals^13,18^. WAS also counteracts behavioural disturbances in animal models of IBD^9,61^. In contrast, the effects of WAS on microglial phenotype is unknown and thus we examined whether acute WAS alters microglial activation or modifies colitis-induced microglial changes. However, the current data show that WAS does not affect brain Iba-1 or CD68 levels both during colitis or control conditions. This is surprising, given that microglial activation occurs in response to various stressful incidents including acute water-immersion restraint stress^28^, cold stress^30^, repeated restraint stress^29,54,62^ and prenatal stress^62^. It is, however, possible that a single 30-min WAS session is too mild to change microglial phenotype. Future work will need to look at other microglial markers, which could well be altered by WAS, although Iba-1 and CD68 are not.

In conclusion, our results indicate that murine gastrointestinal inflammation is propagated to the brain causing immunological responses in the form of microglial alterations throughout the limbic system. Given that mice suffering from DSS- or TNBS-induced colitis show a number of behavioural disturbances^11,27,63^, it is intriguing to investigate whether these behavioural changes are actually caused by microglia using a range of pharmacological and genetic tools. The existence of a similar mechanism in IBD patients would be helpful to better understand CNS-immune interactions and the increased prevalence rates of psychiatric disorders such as anxiety disorders and major depression in these patients.

## Methods

### Experimental animals and housing conditions

The study was performed with 68 adult male C57BL/6 N mice obtained from Charles River (Sulzfeld, Germany). The mice were housed under controlled conditions of temperature (set point 21 °C) and air humidity (set point 50%) and under a 12 h light/dark cycle (lights on at 6:00 h, lights off at 18:00 h). The mice were habituated to the animal facility for 2 weeks before any intervention. All experiments were approved by the ethical committee at the Federal Ministry of Science and Research of the Republic of Austria (BMWF-66.010/0119-II/3b/2011 and BMWFW-66.010/0146-V/3b/2018) and conducted according to the Directive of the European Parliament and of the Council of 22 September 2010 (2010/63/EU).

### Study design

This work included three experiments:

In the first experiment, 32 mice were equally allocated to 2 treatment groups (n = 16/group). They either received 2% DSS (molecular weight 36,000–50,000; MP Biomedicals, Illkirch, France) added to the drinking water to induce mild colitis or tap water (control group). After 7 days of treatment, both groups were further subdivided into stressed (30-minute session of WAS) and unstressed animals (4 groups, n = 8/group, Table 1). Ninety minutes post-WAS, the animals were euthanized with an overdose of pentobarbital (150 mg/kg injected intraperitoneally).

In the second and third experiment, 16 and 20 animals were allocated to 2 groups (control group and DSS group), respectively. Like in experiment 1, animals were euthanized after 7 days of treatment.

After euthanasia, brains were collected, frozen on dry ice, and processed for immunohistochemistry (experiment 1), qPCR (experiment 2) or flow cytometry (experiment 3). Colitis was assessed by measuring DAI on the one hand and colonic levels of MPO on the other hand.

### Disease activity index

The DAI was used to evaluate the physical status of the animals and was calculated using 3 variables as previously described^11^: weight difference between start and end of DSS treatment (score 0: weight increase ≥1 g, score 1: weight increase <1 g, score 2: weight decrease <1 g, score 3: weight decrease ≥1 g), stool consistency (score 0: normal stool, score 1: soft but formed stool, score 2: loose stool) and presence of blood in the perianal region (score 0: no traces of blood in the perianal region, score 1: traces of blood in the perianal region, score 2: bloody perianal region). Accordingly, the minimum score was 0 and the maximum score was 7^11^.

### Colonic myeloperoxidase content

Colonic MPO content reflects infiltration of the colonic tissue with leucocytes and macrophages^19^. After euthanasia, full-thickness specimens of the distal colon were excised, shock-frozen in liquid nitrogen and stored at −70 °C until MPO assay (Hycult Biotechnology, Uden, The Netherlands). Briefly, tissues were weighed and homogenized in lysis buffer (200 mM NaCl, 5 mM ethylenediaminetetraaceticacid, 10 mM trishydroxymethylaminomethane, 10% (v/v) glycerine,1 mM phenylmethylsulphonylfluoride (PMSF, Sigma-Aldrich P7626), 1 mg/ml leupeptide, and 28 mg/ml aprotinin, pH7.4) at a tissue:lysis buffer ratio of 1 mg:0.02 ml. Tissue debris was pelleted by centrifugation (6000 × g, 4 °C, 15 min) and the supernatant was used for enzyme-linked immunosorbent assay according to the manufacturer’s instructions.

### Water avoidance stress

The WAS procedure was carried out in a brightly lit room (230–250 lux). Mice were placed on a small platform (6 × 3 × 6 cm, length × width × height) in the centre of a water-filled box (61 × 40 × 22 cm, length × width × height), the level of the water (25 °C) in the box being 0.5 to 1 cm below the platform as previously described^9,11,13^. Following exposure to WAS for 30 min, the animals were returned to their home cage. Ninety minutes post-WAS the animals were euthanized with an overdose of pentobarbital (150 mg/kg injected intraperitoneally).

### Immunohistochemistry

Immunohistochemistry procedures were performed as described previously with minor modifications^13,64^. Briefly, fresh frozen brains were sectioned at 20 µm thickness, mounted on Superfrost Plus slides (Menzel, Braunschweig, Germany) and fixed in 4% paraformaldehyde (Sigma-Aldrich, Vienna, Austria) in PBS. Endogenous peroxidases were quenched with 0.3% H_2_O_2_ in methanol and 10% normal goat serum in antibody diluent (v/v) was used for protein blocking. Sections were incubated with the primary antibodies rabbit anti-Iba-1 (1:1000) (Wako Chemicals, Neuss, Germany) or rabbit anti-CD68 (1:1000) (ab125212, abcam, Cambridge, UK) in antibody diluent (AD) (0.1 M PBS containing 0.05% Tween 20, 2% normal goat serum and 1% bovine serum albumin) overnight at 4 °C and for 30 min in AD containing the biotinylated secondary antibody (goat anti-rabbit IgG 1:200) (Vectastain Elite ABC Kit, Vector Laboratories, Burlingame, USA) at room temperature. After incubation with avidin-biotin complex solution (Vectastain Elite ABC Kit), sections were developed with 3, 3-diaminobenzidine (DAB substrate kit for peroxidase, Vector Laboratories).

### Image analysis and quantification

The immunohistochemically processed brain sections were examined with a light microscope coupled to a computerized image analysis system (MCID; Basic version 7.0, Imaging Research Inc., Brock University, St. Catharines, Ontario, Canada). Slides were coded such that the investigator was blind to the treatment group under investigation. Brain regions of interest (ROIs) were identified with the help of adjacent Nissl-stained sections and the Paxinos and Franklin’s mouse brain atlas^65^. In order to quantify the surface area of all Iba-1 or CD68 positive signals within a ROI (cells and cellular processes containing a brown/black reaction product of sufficient intensity) with the computerized image analysis system, an intensity-based background threshold was determined for each brain ROI. The background threshold was defined such that the maximum number of labelled cells and cell processes was identified without inclusion of any background staining. A 300 × 300 µm counting frame was placed in each ROI to quantify Iba-1 or CD68 signals. Exceptions were the granular cell layer of the DG for both markers and the PVH for Iba-1 where immunoreactive surface area was quantified across the whole structure. Two to three consecutive bilateral sections were evaluated in each ROI for each mouse (8 mice used per group) and the average values/brain region of each animal were used for statistical analysis.

### Brain microdissection procedure

The brain microdissection of the mPFC was performed as described previously^66^. After cleaning the working area and dissection instruments with RNase AWAY (Carl Roth, Karlsruhe, Germany), the brains were cut into 1 mm thick slices at −20 °C in the cryostat. Then the mPFC (Bregma, +3.20 to −0.22) was micro-dissected under a stereomicroscope on a cold plate (Weinkauf Medizintechnik, Forchheim, Germany) at −20 °C. The dissected mPFC was collected in micro packaging vials filled with Precellys beads (Peqlab, Erlangen, Germany) and stored at −70 °C until RNA extraction.

### RNA extraction and RT-qPCR

RNA extraction and qPCR were performed as described previously^67^. Brain tissues were homogenized with a Precellys 24 homogenizer (Peqlab Biotechnology, Polling, Austria) in QIAzol reagent and RNA was extracted with the RNeasy lipid tissue mini kit (Qiagen, Germany). 1 µg of the RNA solution was reverse-transcribed using the high capacity cDNA reverse transcription kit (Applied Biosystems, Foster City, California, USA). A no RT control for each sample was also included. For relative quantification of mRNA levels, qPCR was performed on a CFX384 Touch™ Real-Time PCR Detection System (BioRad, Vienna, Austria). TaqMan gene expression master mix (Life Technologies, Carlsbad, CA, USA) was used in the assay along with specific TaqMan primers (Thermo Fischer Scientific, Waltham, MA, USA). The measured targets included: Iba-1 (Mm00479862_g1), CD68 (Mm03047343_m1), CD11b (Mm00434455_m1), CD86 (Mm00444543_m1), CD206 (Mm01329362_m1), IL-1β (Mm00434228_m1), TNF-α (Mm00443258_m1), Nos2 (Mm00440502_m1), Arg1 (Mm00475988_m1), Chil3 (Mm00657889_mH) and IDO-1 enzyme (Hs00158027_m1). All samples were measured as triplicates. ACTB (Mm00607939_s1) and GAPDH (Mm99999915_g1) were used as reference genes for quantification of target gene expression. Quantitative measurements of target gene levels relative to controls were performed with the ΔΔ Cq method using the mean value of the control group as the calibrator. Group differences were expressed as fold changes.

### Flow cytometry

Single cell suspensions were obtained as described previously with minor modifications^68^. Animals were sacrificed, and their brains were removed from skulls immediately after cardiac perfusion with 10 mL of ice-cold PBS. Mouse brains were minced using scissors and mechanically dissociated and gently forced through a 100-μm strainer followed by rinsing with 20 mL of PBS plus 2% FBS (Staining Buffer, SB). Cells were pelleted by centrifugation (500 g for 8 min at 4 °C) and suspended in red blood cell (RBC) lysis buffer (Biolegend, California, U.S.) (2 mL/brain, 5 minutes on ice). RBC lysis was quenched by adding 3 volumes of SB, and cells were centrifuged at 500 g for 8 minutes at 4 °C. To remove myelin, cells were suspended in 10 mL of 30% Percoll (Sigma-Aldrich) in Hanks’ balanced salt solution (HBSS, Life Technologies) and centrifuged at 700 g for 10 minutes at room temperature without break. The myelin layer at the top was removed, and the cell pellet was suspended in SB to perform cell counts and FACS staining. Cells were incubated for 10 min in Fc receptor blocking solution (1,5 μL Fc-block + 48,5 μL SB per test, Biolegend) and stained with fluorochrome-conjugated anti-mouse antibodies against: CD45 (APC-CD45 Biolegend, 103112) and CD11b (PECy7-CD11b, BD Biosciences, 552850) for 30 min. Cells were washed two times with 200 μL of SB, suspended in 100 µl of SB + 2 µl of propidium iodide (BD Biosciences, California, U.S.) and measured immediately on a Canto II flow cytometer with FACSDiva software (BD Biosciences). Compensation and analysis were performed using FlowJo software (Tree Star, Oregon, U.S.), and gates were defined by fluorescence minus one (FMO) controls.

## Data Availability

All data generated or analysed during this study are included in this published article or available from the corresponding author on reasonable request.
